# Cadaveric and three-dimensional computed tomography study of the morphology of the scapula with reference to reversed shoulder prosthesis

**DOI:** 10.1186/1749-799X-3-49

**Published:** 2008-10-10

**Authors:** Carlos Torrens, Monica Corrales, Gemma Gonzalez, Alberto Solano, Enrique Cáceres

**Affiliations:** 1 Orthopaedic Department, Hospital del Mar de Barcelona, Passeig Marítim 25-29, 08003 Barcelona, Spain; 2 Department of Radiology, Hospital del Mar de Barcelona, Passeig Marítim 25-29, 08003 Barcelona, Spain

## Abstract

**Purpose:**

The purpose of this study is to analyze the morphology of the scapula with reference to the glenoid component implantation in reversed shoulder prosthesis, in order to improve primary fixation of the component.

**Methods:**

Seventy-three 3-dimensional computed tomography of the scapula and 108 scapular dry specimens were analyzed to determine the anterior and posterior length of the glenoid neck, the angle between the glenoid surface and the upper posterior column of the scapula and the angle between the major craneo-caudal glenoid axis and the base of the coracoid process and the upper posterior column.

**Results:**

The anterior and posterior length of glenoid neck was classified into two groups named "short-neck" and "long-neck" with significant differences between them. The angle between the glenoid surface and the upper posterior column of the scapula was also classified into two different types: type I (mean 50°–52°) and type II (mean 62,50°–64°), with significant differences between them (p < 0,001). The angle between the major craneo-caudal glenoid axis and the base of the coracoid process averaged 18,25° while the angle with the upper posterior column of the scapula averaged 8°.

**Conclusion:**

Scapular morphological variability advices for individual adjustments of glenoid component implantation in reversed total shoulder prosthesis. Three-dimensional computed tomography of the scapula constitutes an important tool when planning reversed prostheses implantation.

## Background

The anatomy of the scapula has been descriptively studied taking into account the anthropometric measurements and geometry [1-5], but recently several studies have focused the study of the scapula to better understand and manage pathomechanics of instability [6-10], cuff disorders and snapping scapula [11]. Anatomic total shoulder replacement has also been the subject of radiological and tomographic scapular anatomic studies to better understand biomechanics and component implantation [12-16]. Reversed shoulder prosthesis have been proved to be successful for the treatment of painful glenohumeral arthritis associated with an irreparable rotator cuff tear at least at short and mid-term follow-up [17-20]. Biomechanical studies support the benefit of the reversed prosthesis design in front of anatomical designs when there is a complete loss of rotator cuff function [21]. However some studies have advised the potential source of problems the reversed design can produce [22,23]. The major concern is referred to glenoid component loosening. In the Delta III reversed prosthesis (DePuy International Ltd, Leeds, England), the glenoid component is fixed to the glenoid trough a central peg that should be located into the glenoid body and four screws to be located in the base of the coracoid process, the upper posterior column of the scapula and the body of the glenoid respectively. It is supposed that the better the peg and screws are placed, the best primary fixation will be obtained [24].

The purpose of this study is to analyze the morphology of the scapula with reference to the glenoid component implantation in reversed shoulder prosthesis, in order to improve primary fixation of the component.

## Methods

Seventy-three consecutive 3-dimensional computed tomography of the scapula obtained from the image studies of 52 patients with proximal humeral fractures and 21 patients with recurrent antero-inferior instability were included. Mean age of the whole serie was of 52.59 years old (ranging from 16 to 84). There were 46 females and 27 males. A digitalized true anterior view, a true posterior view and a profil view of the scapula were obtained from each patient. To obtain reproducible images from all the 3-D reconstructed scapulas, true anterior and posterior views were obtained by rotating the reconstructed 3-D image through the craneo-caudal axis until the glenoid surface appeared as a simple line and rotating then this image through the lateral to medial axis until the inferior part of the coracoid process reach the upper part of the glenoid in the anterior view and until the acromion reach the upper part of the glenoid in the posterior view. Glenoid version was measured in the two populations of patients studied by 3-dimensional computed tomography (instability group and fracture group) without significant differences between them (instability group mean glenoid retroversion of 4°, ranging from 5° of anteversion to 18° of retroversion, and fracture group mean glenoid retroversion of 6°, ranging from 3° anteversion to 22° of retroversion). The following measures were made on each patient: length of the neck of the inferior glenoid, angle between the glenoid surface and the upper posterior column of the scapula, angle between the major craneo-caudal glenoid axis and the base of the coracoid process and angle between the major craneo-caudal glenoid axis and the upper posterior column of the scapula. The length of the neck of the inferior part of the glenoid was measured in the true anterior view as well as in the true posterior view. The length of the neck of the glenoid was measured at its inferior part through the index formed by the craneo-caudal glenoid surface measure and the distance from the inferior angle of the glenoid surface to the anterior and posterior columns of the scapula. The angle between the glenoid surface and the upper posterior column of the scapula was measured in the true posterior view.

**Figure 1:**
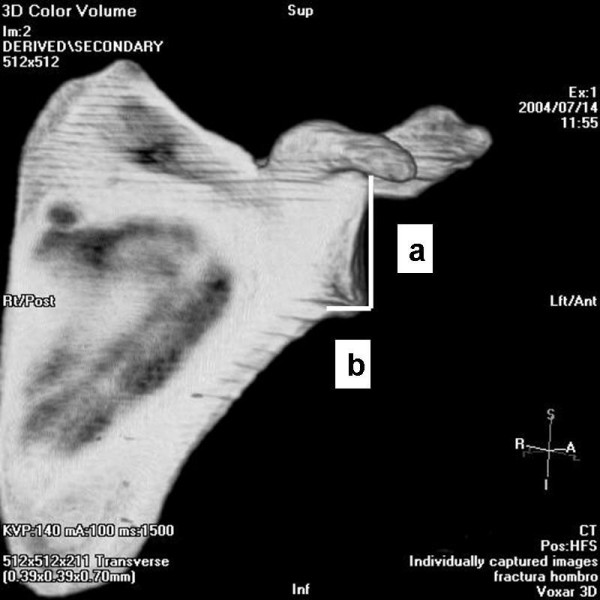
**Anterior measure of the inferior glenoid neck index.** a, articular glenoid surface measure; b, distance from articular glenoid surface to anterior and posterior column of the scapula.

**Figure 2:**
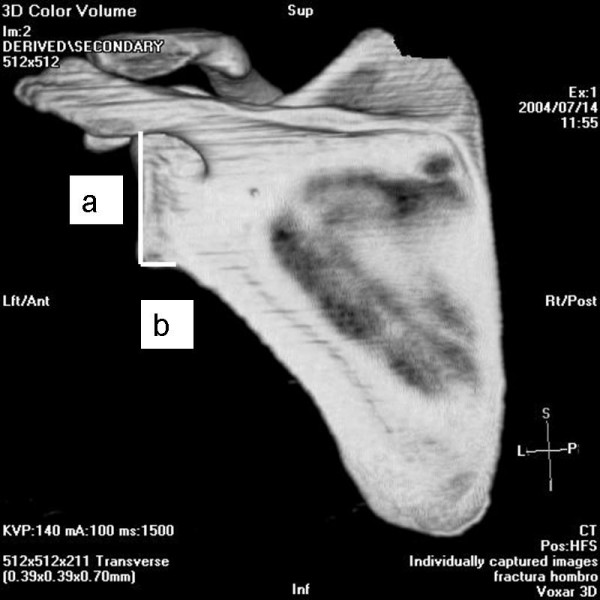
**Posterior measure of the inferior glenoid neck index.** a, articular glenoid surface measure; b, distance from articular glenoid surface to anterior and posterior column of the scapula.

**Figure 3:**
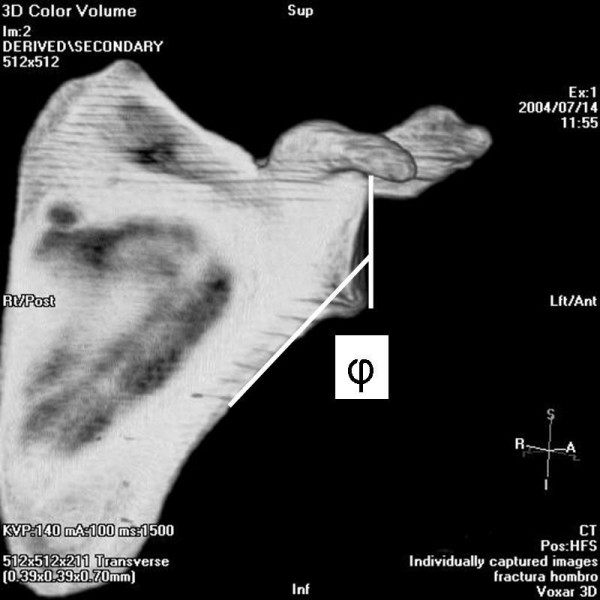
Measure of the angle between the glenoid surface and the upper posterior column of the scapula (φ).

**Figure 4:**
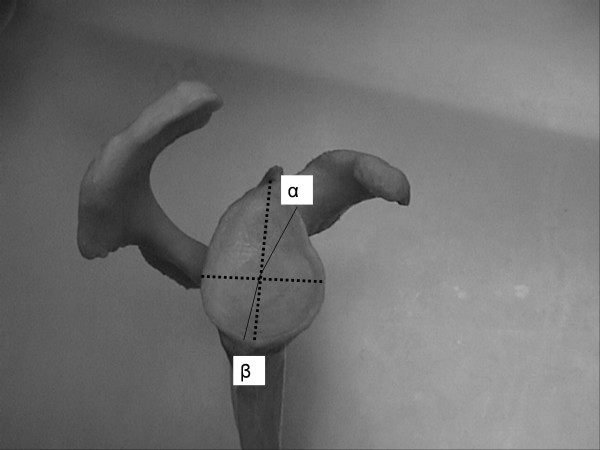
Measure of angle between the major craneo-caudal glenoid axis and the base of the coracoid process (α) and angle between the major craneo-caudal glenoid axis and the upper posterior column of the scapula (β).

The angle between the major craneo-caudal glenoid axis and the base of the coracoid process and the angle between the major craneo-caudal glenoid axis and the upper posterior column of the scapula were measured in the outlet view of the scapula (Figure 1, Figure 2A–B, Figure 3 and Figure 4). All measures were digitally performed.

One-hundred-eight scapular dry specimens, obtained from the Anatomy Collection of Skeletons at Medicine University of Barcelona and Medicine University of Madrid, were examined. No epidemiological data was available for the specimens. Because specimens were collected at the Anatomy Department of two different Universities it was not possible to obtain C.T. scans and digitalized images, so all measures were manually performed. The length of the neck of the inferior part of the glenoid was measured in the anterior as well as in the posterior faces of the glenoid. The angle between the glenoid surface and the upper posterior column of the scapula was measured in the posterior face of the glenoid. All measures were manually performed with the aid of a goniometer and a caliper and were directly performed to bone by placing the caliper at the more inferior part of the glenoid and by directly applying the goniometer to the glenoid surface and upper posterior column of the scapula. Because the measures were manually done and drawing lines in the specimens was not allowed no attempt was made to measure the angles on the profile view.

Two observers independently performed all the measures twice in the digitalized images to allow inter and intraobserver studies to be done. Scapular dry specimens were also measured independently by two observers to allow interobserver study. This studies were analyzed through the Kappa index.

Statistics included Mann-Whitney U test andd *x*^2 ^test. Significance was defined at p < 0.05.

## Results

**Figure 5:**
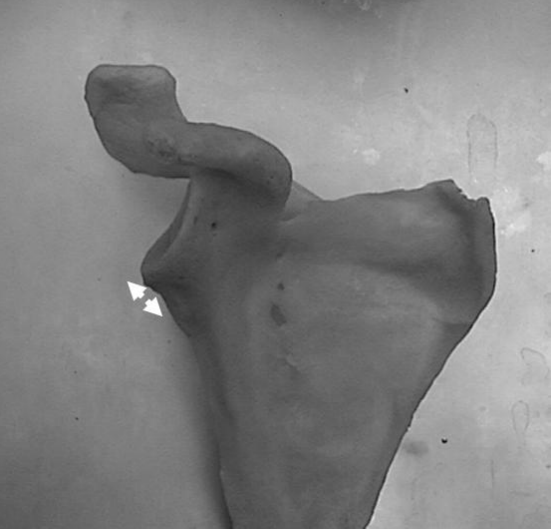
Anterior short neck glenoid.

**Figure 6:**
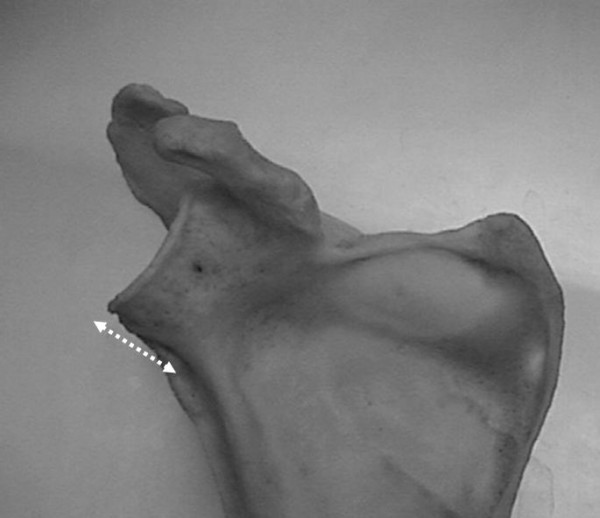
Anterior long neck glenoid.

Both three-dimensional computed tomography scapulas and cadaveric scapulas were divided into two different groups according to the length of the neck of the glenoid because they belonged to two different clusters, the one named "short-neck" and the other named "long-neck". Mean index of length in the "short-neck" group was of 3,12 (ranging from 2,66 to 4,20) for the three-dimensional computed tomography scapulas while in the cadaveric group was of 3,24 (ranging from 2,29 to 3,36). Mean index of length in the "long-neck" group was of 2,27 (ranging from 1,94 to 2,52) for the three-dimensional computed tomography scapulas while in the cadaveric group was of 2,35 (ranging from 2,00 to 2,73). The "short-neck" group represented the 41,82% in the three-dimensional computed tomography scapulas and the 18,27% in the cadaveric group while the "long-neck" represented the 58,18% and the 81,73% respectively. There were statistically significant differences between both groups (p < 0,001 for the three-dimensional computed tomography scapulas with a 95% CI of 0,002–0,45 and p = 0,034 for the cadaveric group with a 95% CI of 0,25–0,79). (Figure 5 and Figure 6)

The length of the neck of the posterior glenoid was also classified into two groups named "short-neck" and "long-neck" for both three-dimensional computed tomography and cadaveric scapulas. Mean index of length in the "short-neck" group was of 4,80 (ranging from 4,22 to 5,41) for the three-dimensional computed tomography scapulas while in the cadaveric group was of 4,00 (ranging from 3,70 to 4,53). Mean index of length in the "long-neck" group was of 3,84 (ranging from 3,09 to 4,54) for the three-dimensional computed tomography scapulas while in the cadaveric group was of 3,58 (ranging from 3,12 to 4,13). The "short-neck" group represented the 34,48% in the three-dimensional computed tomography scapulas and the 59,80% in the cadaveric group while the "long-neck" represented the 65,51% and the 40,20% respectively.

**Figure 7:**
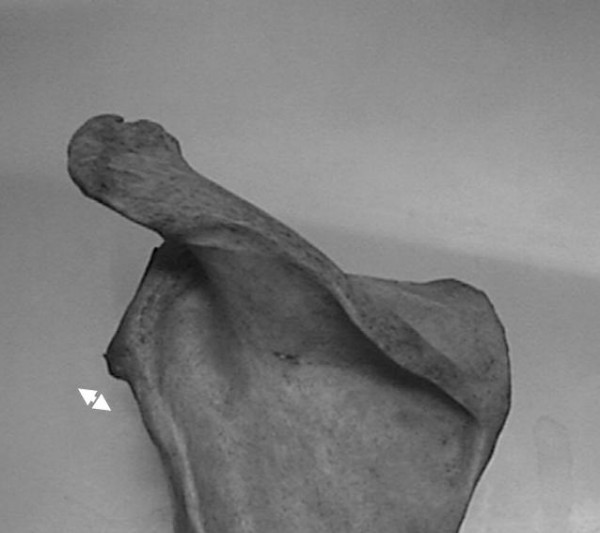
**A: Posterior short neck glenoid.** B: Posterior long neck glenoid.

**Figure 8:**
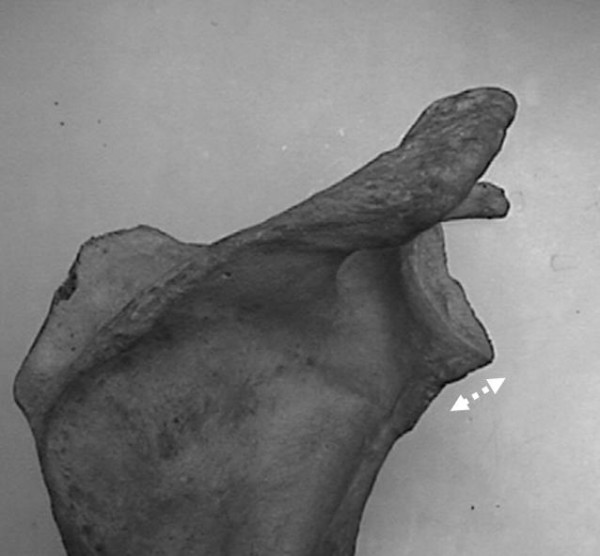
Posterior long neck glenoid.

There were statistically significant differences between both groups (p = 0,002 for the three-dimensional computed tomography scapulas with a 95% CI of -0,89 and 0,04 and p = 0,020 for the cadaveric group with a 95% CI of 0,4–0,95).(Figure 7 and Figure 8) Table 1

**Table 1:** 3-D CT and Specimen values of anterior and posterior glenoid neck length

	Ant "short-neck"	Ant "long-neck"	p value	Post "short-neck"	Post "long-neck"	p value
3-D CT	3,12 (2,66–4,2)	2,27(1,94–2,52)	p < 0,001	4,8(4,22–5,41)	3,84(3,09–4,54)	p = 0,002
Specimen	3,24(2,29–3,36)	2,35(2–2,73)	p = 0,034	4(3,70–4,53)	3,58(3,12–4,13)	p = 0,020

**Figure 9:**
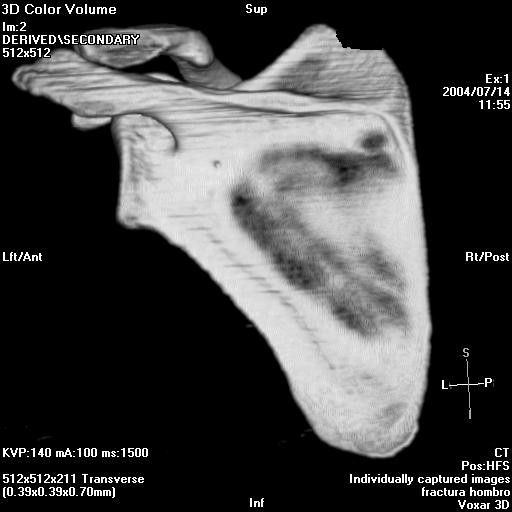
**Type I angle between the glenoid surface and the upper posterior column of the scapula.** B: Type II angle between the glenoid surface and the upper posterior column of the scapula.

**Figure 10:**
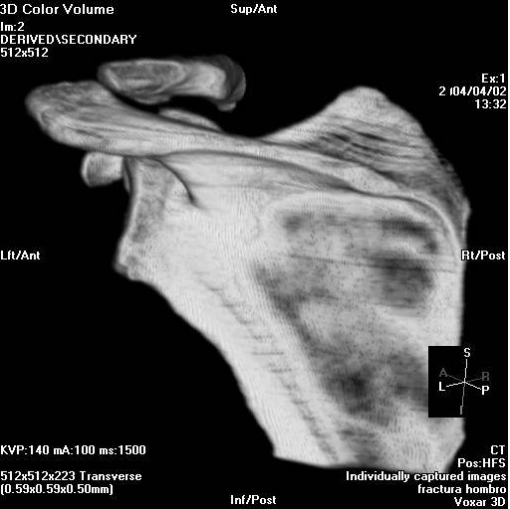
Type II angle between the glenoid surface and the upper posterior column of the scapula.

The angle between the glenoid surface and the upper posterior column of the scapula was also classified into two different types: type I and type II. Mean type I angle was of 52° (ranging from 48° to 57°) for the three-dimensional computed tomography scapulas while in the cadaveric group were of 50° (ranging from 49,25° to 55°). Mean type II angle was of 64° (ranging from 60° to 70°) for the three-dimensional computed tomography scapulas while in the cadaveric group was of 62,50° (ranging from 60° to 66,75°). Type I represented the 61,43% in the three-dimensional computed tomography scapulas and the 71,30% in the cadaveric group while type II represented the 38,57% and the 28,70% respectively. There were statistically significant differences between both groups (p < 0,001 for the three-dimensional computed tomography scapulas with a 95% CI of -5,53 and -1,17 and p < 0,001 for the cadaveric group with a 95% CI of -14,67 and -10,31).(Figure 9) and Figure 10. 

The angle between the major craneo-caudal glenoid axis and the center of the base of the coracoid process averaged 18,25° (ranging 13° from to 27°). The angle between the major craneo-caudal glenoid axis and the upper posterior column of the scapula averaged 8° (ranging 5° from to 18°). Table 2

**Table 2:** 3-D CT and Specimen values of the angle between the glenoid surface and the upper posterior column of the scapula and the angle between the major craneo-caudal glenoid axis and the center of the base of the coracoid process and the upper posterior column of the scapula

	Type I	Type II	p value	Coracoid	post column
3-D CT	52°(48°–57°)	64°(60°–70°)	p < 0,001	18,25° (13°–27°)	8° (5°–18°)
Specimen	50°(49°–55°)	62,5°(60°–66,75°)	p < 0,001	-	-

No differences could be found between anterior glenoid neck length, posterior glenoid neck length, type I or II angle of glenoid surface and posterior column of the scapula regarding sex and age in the three-dimensional computed tomography patients studied.

Intraobserver analysis of the anterior glenoid neck length gave a Kappa index of 0,655 and 0,661 respectively for each observer, the posterior glenoid neck length of 0,503 and 0,629 and the type of angle of glenoid surface and upper posterior column of the scapula of 0,831 and 0,889. Interobserver analysis of the anterior glenoid neck length gave a Kappa index of 0,518, the posterior glenoid neck length of 0,398 and the type of angle of glenoid surface and upper posterior column of the scapula of 0,470.

## Discussion

Anatomic studies have moved from simply descriptive [1-5] to pathomechanical explanation of several shoulder disorders and to specific surgical techniques development.

Recently, reversed shoulder prosthesis design has gained popularity in the management of massive cuff tears associated with glenohumeral arthritis, even though results refer short and mid term follow-up [17-20]. The reversed design is, however, cause of concern because of the fixation of its components, specially the glenoid component, as well as the potentially rate of complications such as component loosening [22,23]. When this study was carried out, Delta III (DePuy International Ltd, Leeds, England) was the unique reversed shoulder prostheses available in Spain. Primary fixation of the glenoid component in Delta III prosthesis relays on a central stem that should be located into the glenoid body, and four screws. Delta III glenoid component present a fixed – angle orientation of the superior and inferior screws (70° between glenoid surface and screw) and a free-angle orientation for the anterior and posterior ones. Superior and inferior screws should be located in divergence, directing the superior one to the base of the coracoid process and the inferior one to the upper posterior column of the scapula. The anterior and the posterior screws should be placed into the body of the glenoid. In addition, the superior and inferior holes of the glenoid component to insert the superior and the inferior screws are positioned in line. It is to be supposed that fail in peg and/or screws location may affect stability of the implant as it has been shown in previous studies [24]. It is also to be supposed that the more the screws run inside the bone, the better fixation will be obtained.

The present study has found two different types of scapulas as far as glenoid surface to upper posterior column of the scapula angle is concerned, and although no attempt has made to measure the 3-D bone coverage of the inferior screw in the different types of scapulas, type I, which is the most frequent (61,43% in the three-dimensional computed tomography scapulas and the 71,30% in the cadaveric group), determines a mean angle of 50°–52°, meaning that if the inferior screw has a prefixed position of 70°, it will be poorly placed into bone because the different orientation of the screw and the lateral border of the scapula determining thus less bony coverage. Type II determines a mean angle of 62°–64°, meaning that the prefixed screw direction better fits in the lateral border of the scapula leading to a more bony coverage of the screw.

Taking into account the coronal plane, this study demonstrates that the center of the coracoid process and the upper posterior column of the scapula are not in line, moreover, the center of the base of the coracoid process is located a mean of 18,25° anterior with regard to the major craneo-caudal glenoid axis and the upper posterior column of the scapula is located 8° posterior to this axis, giving a mean of 10°of difference. In the Delta III glenoid component the holes for the superior and inferior screws are placed in line, that means that if the inferior screw is properly located in the posterior column of the scapula, the superior screw is directed to the posterior part of the base of the coracoid process, giving thus a poor placement into bone.

The inferior part of the glenoid in the anterior face as well as in the posterior can be divided into two grossly different length necks. In the so called "short-length" glenoid neck, the glenoid articular surface is close to the upper posterior column of the scapula and allows inferior screw to reach easily to the posterior column of the scapula. In the so called "long-neck" glenoids, the glenoid articular surface is located far from the upper posterior column of the scapula and determines that if the inferior screw has a prefixed angle it may conduct the screw through the glenoid neck instead of into the upper posterior column of the scapula, giving thus a short bone in through location.

All the anatomical variations described advice for major changes in the metaglene component of the reversed prostheses to improve bone fixation. Inferior and superior screws may have to have a minimum of 10° of free orientation to adapt in the upper part of the posterior column of the scapula and be able to fit both scapular types. The 10° free orientation may also help to better place the superior screw into the base of the coracoid process.

One major cause of concern regarding the glenoid component fixation is the formation of a notch at the inferior pole of the scapula as a result of the contact of the medial part of the humeral component and the glenoid during adduction. Recently, to avoid this complication, the implantation of the glenoid component extending beyond the inferior glenoid rim has been proposed [25]. Several preoperative measures have to be done before deciding to extend beyond the inferior glenoid rim the glenoid component to assess the type of scapula and the length of the inferior glenoid neck. Positioning inferiorly the glenoid component in case of a "long-neck" glenoid may determine the screw run through the glenoid neck instead of into the upper posterior column, and in the same way, in a type I scapula the more inferior the glenoid component is located, the less chance to get the lateral border of the scapula with the inferior screw in an angle-fixed component design. Avoiding scapular notch by extending beyond the inferior glenoid rim the glenoid component positioning requires glenoid component to be modified in order to allow variation in the direction of positioning the inferior screw.

The different scapular morphologies founded in this study advise to individualize screws positioning in the glenoid component to adjust them to the anatomy present in each particular case. Three-dimensional computed tomography of the scapula constitutes an unvalued source when planning surgery with reversed prostheses for better understanding the particular scapular morphology of each individual case and the adjustments to be done to better place glenoid component. Prefixed angle screws leads several times to a decrease of bone coverage, so adjustments have to be done to change direction depending on the type of angle between the glenoid surface and the upper posterior column of the scapula, the different location of the base of the coracoid process and the upper posterior column of the scapula and the length of the neck of the glenoid. Maybe two different implant types of glenoid component should be considered to address different glenoid neck lengths.

Recently Codsy et al have also stressed on the importance of the glenoid vault and the integrity of the subchondral bone to obtain proper fixation of the glenoid component and even though they find in normal glenoids a uniform morphology of the glenoid vault, 5 different sizes are defined to fit an average clinical population.

Is to be believed that bony coverage of the screw may affect stability if the implant although many other parameters are involved in glenoid component stability such as bone quality around screw, orientation of the screw with respect to the forces, etc.

No relationship has been found between the different scapular morphologies and sex or age in the three-dimensional computed tomography group. No correlation has been found between the different types of scapulas as far as glenoid surface and posterior column of the scapula angle is concern and glenoid neck length in anterior or posterior face. No correlation has been found between the length of the neck in the anterior face of the glenoid and the length of the neck in the posterior face.

Kappa studies revealed a moderate to substantial agreement of anterior and posterior neck lengths which means a reasonable level of concordance and reproducibility of these measures, and a level almost perfect in the analysis of the type of angle of glenoid surface and upper posterior column of the scapula.

## Conclusion

Scapulas can be classified into two groups regarding the angle between the glenoid surface and the upper posterior column of the scapula with significant differences between them, two different lengths of the neck of the inferior glenoid body have also been differentiated in the anterior as well as in the posterior faces of the scapula, and finally the base of the coracoid process is not in line with the posterior column of the scapula. Good concordance and reproducibility as showed by kappa studies.

All the scapular morphologic variability described advice for individual adjustments of glenoid component implantation in Delta III reversed total shoulder prosthesis. Three-dimensional computed tomography of the scapula constitutes and important tool when planning reversed prostheses implantation.

## Competing interests

The authors declare that they have no competing interests.

## Authors' contributions

CT conceived the study and analized CT scans and cadaveric specimens and drafted the manuscript. MC analized cadaveric specimens and participate in Kappa study. GG analized CT scans and participate in Kappa study. AS prepared CT images, 3-D images and analized them. EC participate in the conception of the study participated in its design and coordination. All authors read and approved the final manuscript.
